# Teaching and Learning of Clinical Competence in Ghana: Experiences of Students and Post-Registration Nurses

**DOI:** 10.3390/healthcare10030538

**Published:** 2022-03-15

**Authors:** David Abdulai Salifu, Yolande Heymans, Christmal Dela Christmals

**Affiliations:** 1 Centre for Health Professions Education, Faculty of Health Sciences, North-West University, Potchefstroo Campus, Building PC-G16, Office 101,11 Hoffman St., Potchefstroom 2520, South Africa; dsalifu@uds.edu.gh (D.A.S.); yolande.heymans@nwu.ac.za (Y.H.); 2 School of Nursing and Midwifery, University for Development Studies, Box TL 1350, Tamale 00233, Ghana

**Keywords:** clinical competence, clinical nursing education, Ghana, qualitative descriptive, simulation

## Abstract

Despite the growing demand for competent nurses to fill the shortage gap, nursing education institutions have not always been able to equip students with the requisite clinical competence needed in the practice setting. Several studies have described the experiences of undergraduate nursing students in the clinical learning environment. No study was found on the experiences of diploma nursing students and post-registration nurses regarding the teaching and learning of clinical competence in Ghana. This study, therefore, sought to describe the experiences and perceptions of diploma nursing students and post-registration nurses regarding the teaching and learning of clinical competence in Ghana. A qualitative descriptive research design was employed in this study. Fifty-five (55) participants, comprising 40 students and 15 post-registration diploma nurses, from six research sites were recruited into focus group discussions (FGDs) using a maximum variation purposive sampling technique. A thematic framework method was used to analyze the data with the aid of ATLAS.ti software. Three themes emerged from the focus group discussions: nursing education institutional factors; clinical placement design, implementation, and system challenges; challenges of clinical teaching and learning. We conclude that the current approach to clinical nursing education, such as the overreliance on clinical placement and the use of more teacher-centered teaching approaches, are ineffective in facilitating the development of clinical competence. A review of the nursing curriculum, inculcating evidence-based simulation modalities, and an adequate investment in nursing education may be required to ensure effective nursing education in the study setting.

## 1. Introduction

Clinical education is at the core of nursing education, as nursing is a practice-based profession. Clinical nursing education refers to the strategies adopted by Nursing Education Institutions (NEIs) to ensure that nursing students develop clinical competence, either in the skills laboratory or in real-life clinical settings, in order to meet the standards set by nursing councils for qualification and registration as nurses [1]. Clinical nursing education provides the platform for nursing students to actualize theory in direct patient care activities and, by so doing, be socialized to the real-world demands of the nursing profession [2]. Therefore, a critical link exists between the quality of clinical nursing education experience and the development of clinical competence. Clinical nursing competence refers to the ability to integrate knowledge, experience, clinical reasoning, sound clinical judgement, skills, beliefs, values, and attitudes into the fulfilment of one’s professional role as a nurse in a given situation [3,4,5].

To promote the development of clinical competence, nursing education is designed to inculcate both theoretical and practical content that usually occur in NEIs and/or clinical practice settings (CPSs) [6,7]. As a practice-based profession, the essence of the practical aspect of the curriculum cannot be overemphasized [6,7]. Because of the need to provide nursing students with optimum practice opportunities in CPSs, the clinical placement hours of nursing students are one of the highest among healthcare professions [8]. Despite the centrality of competence in nursing education, certain observations in the CPSs have called into scrutiny the clinical competence of nurses. For example, there are the consequences associated with the late recognition of physiological deterioration among hospital in-patients, medication errors, and poor aseptic techniques are numerous [6,8,9]. Although perceived insufficient clinical competence is considered a problem with nurses in general, it is more prevalent among nursing students and immediate post-registration nurses [10]. Consequently, immediate post-registration nurses experience certain difficulties while transitioning into full-time professional roles, leading to poor job satisfaction and high job attrition [11,12]. 

The literature is replete with explanations for the lack of clinical competence demonstrated by nurses upon joining the health workforce [8,13,14,15]. Nurse educators of NEIs have been blamed for the use of outdated, non-standardized clinical teaching methods and materials [13,16], calling into question the expertise of persons recruited to teach in NEIs. Botma et al. [17] espoused the need for well-trained nurse educators and clinicians who work in an environment of mutual trust characterized by the use of student-centered teaching strategies as critical in developing clinical competence. Sadly, Salifu et al. [13] found that most nurse educators lacked the qualification and clinical expertise needed to teach in NEIs. Moreover, most NEIs in low-resource settings lack the necessary infrastructure and resources to support student learning [13,18]. Consequently, NEIs resort to placing students in clinical settings to enhance their clinical competence [19,20]. However, challenges in the CPS, such as the lack of resources and support, and the emergence of pandemics appear to be excessively restrictive, limiting learning opportunities and impeding clinical competence development [13,21].

Consequently, most NEIs have resorted to blending clinical placement with simulation-based clinical nursing education (SBCNE) to ensure effective mastery of clinical competence [22,23,24]. Clinical competence seems to be better developed through the use of experiential and student-centered teaching and learning strategies. Despite the positive impact of SBCNE, it has not been successfully used in low-resource settings, partly due to limitations in the application of frameworks developed in high-income countries [25]. Understanding the issues associated with the development of clinical competence in low-resource settings is necessary to aid in the redesign and implementation of clinical education to facilitate the development of clinical competence. To better understand the experiences of students and post-registration nurses in the teaching and learning of clinical competence in Ghana, we conducted focus group discussions (FGDs) with nursing students and post-registration nurses in six sites across three zones in Ghana. Our aim was to describe the experiences and perceptions of students and post-registration nurses with regards to the teaching and learning of clinical competence in Ghana, a low-resource setting.

### Nursing Education in the Research Context

Nursing education in Ghana began as an apprenticeship program, where recruited novices learned about the job from the British colonial nursing sisters [26]. As the need for more knowledgeable and skillful nurses increased, the Nightingale Fund was instituted for the establishment of a one-year nursing training program in the setting. This training included teaching nurses the theory of disease while they received their practical training from the nursing ‘sisters’ in the hospital wards. Social changes in the 20th century demanded complex nursing services, which resulted in the need to equip nurses with a broader scope of knowledge and skills for practice. This resulted in the establishment of the first nursing college in the capital of the study setting in 1928 [26]. In 1963, the first bachelor’s degree program was established in a university located in the capital town of the setting through the partnership of the government and the World Health Organization. Currently, there are myriads of NEIs across the length and breadth of the setting, implementing general to specialist nursing programs [27]. The majority of the professional nursing workforce in the setting are diploma certificate holders trained by the nursing colleges in spite of the recommendation to make an undergraduate program an entry requirement for nursing [27,28,29]. The training of nurses in the setting has been hampered by the bewildering lack of resources. Infrastructure, logistics, and human resources have all been described as insufficient to satisfy acceptable nursing education requirements [13,27].

## 2. Methods

### 2.1. Design

The study adopted a qualitative descriptive methodology. The approach allowed for a naturalistic inquiry, enabling the description of experiences and perceptions of students and post-registration nurses in the teaching and learning of clinical competence [30]. This approach allowed for the description of the phenomenon in ways that reflected everyday accounts of the phenomenon. 

### 2.2. Study Setting and Participants

The study was conducted in three model nursing colleges (NCs) and their respective primary clinical sites across three geographical zones in Ghana. Second- and third-year nursing students from the three selected NCs and post-registration nurses from the three selected hospitals were recruited for this study using a maximum variation purposive sampling technique. The sampling technique allowed individuals from the various groups to be selected, enabling the identification and description of common and unique experiences among the different groups of participants [31]. Research participants were identified and recruited with the help of independent research administrators at each study site. Eligible students were enrolled in an accredited diploma-awarding public nursing college with a minimum of one-year experience as a student and were fluent in English. Eligible post-registration nurses were in their first year of practice after graduation and similarly fluent in English. Participants who met the inclusion criteria but did not have access to or devices such as computers, tablets, or phones were excluded. Their exclusion was due to financial and logistics constraints of the study.

### 2.3. Data Collection

To ensure homogeneity, FGDs were held separately with students and post-registration nurses. This enabled the free sharing of rich insights from the perspective of participants [32]. Consistent with Gerrish and Lacey’s recommendations [33], a topic guide (Table 1) comprising of 10 open-ended questions, was used in facilitating the FGDs. 

Due to COVID-19, FGDs were held via zoom with the help of an independent focus group facilitator (nurse educator) with experience in facilitating FGDs. We reached saturation after nine sessions of FGDs, when no new information was emerging from the data and the data became repetitious [32]. Each focus group discussion lasted between 60 and 120 min and was conducted in English. With participants’ permission, all FGDs were audio recorded. The data for this study were collected between January and May of 2021.

### 2.4. Ethical Considerations

Ethical approval was granted by the North-West University Health Research Ethics Committee (NWU-00431-20-A1) and the Ghana Health Service Ethics Review Committee (GHS-ERC019/08/20). Participants were briefed in detail about the purpose and procedures of the research through an online information session held via zoom before the signing of a written informed consent form. Each participant was assigned a code to serve as an identifier for confidentiality purposes. The discussions were tape recorded with prior consent of the participants and transcribed verbatim by the first author (D.A.S.). There was no manipulation of participants in the study, and there was no risk of participants being harmed in any way. Participants were told ahead of time that participation in the study was completely optional and that they could opt out at any time with no repercussions.

### 2.5. Data Analysis

FGD data were analyzed using the framework approach of analysis [34] with the aid of ATLAS.ti version 9.0, a qualitative analysis software. Audio recordings of the FGD were transcribed verbatim by D.A.S. and double-checked by a second researcher (C.D.C.) for accuracy. D.A.S. and C.D.C. reviewed the raw data by actively listening to recorded audios and reading the transcripts. They then identified key concepts and themes based on the aim and objectives of the study for detailed examination and referencing of the data. Following this, the transcripts were annotated using numeric codes in ATLAS.ti. D.A.S. and C.D.C. then indexed and charted two scripts independently and compared codes to reach a consensus. The codes were refined and applied by D.A.S. in coding all transcripts. The codes were then grouped into sub-themes and themes. Where the data did not fit into any of the identified themes, new themes were created to accommodate the emerging complexity. This approach allowed for the description of the experiences and perceptions of participants in a way that reflected the everyday perception of the phenomenon.

### 2.6. Rigour

To ensure rigor, the topic guide for the focus group discussions was submitted to an expert in qualitative research and a scholar in teaching and learning for review. Further, the topic guide was pretested in analogous institutions within the same setting with post-registration nurses and nursing students. The topic guide was redesigned after considering feedback from the experts and the pre-test. For example, item 3 was originally item 2 with the phrase “Describe how the skills laboratory is helping you achieve your clinical learning objectives”, but it was renamed item 3 with the phrase “Describe the role the skills laboratory played in helping you achieve your clinical learning objectives”. This was performed to ensure facial legitimacy and clarity. In addition, coding was conducted independently by two study authors (D.A.S. and C.D.C.) and reviewed by a third author (Y.H.) to ensure accuracy. To further enhance the quality of the study, the consolidating criteria for reporting qualitative research (COREQ) checklist was followed [35]. 

## 3. Results

### 3.1. Demographic Characteristics of Participants

Demographic characteristics of study participants are shown in Table 2. A total of nine FGDs were conducted with 55 participants in this study. Participants were made up of twenty second-year and twenty third-year diploma nursing students, as well as fifteen post-registration nurses. 

**Figure 1:**
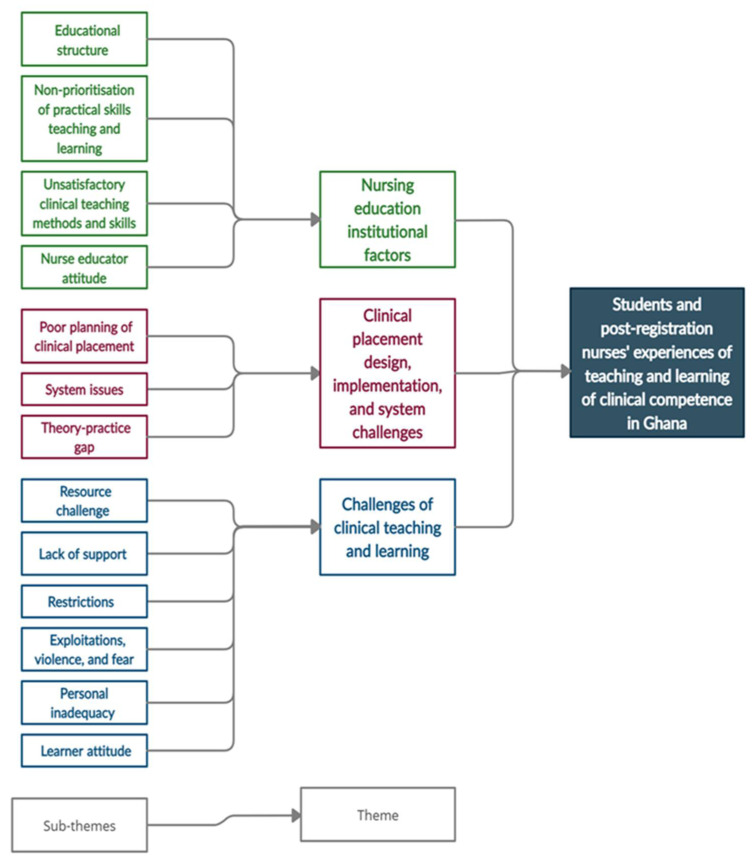
Sub-themes and themes based on students and post-registration nurses’ experiences of teaching and learning of clinical competence in Ghana.

Three main themes emerged from the FGDs: nursing education institutional factors; clinical placement design, implementation, and system challenges; challenges of clinical teaching and learning. Figure 1 illustrates the sub-themes and themes.

Sixty-nine (69) codes were generated, which were then clustered into 15 sub-themes before being regrouped into three main themes: nursing education institutional factors; clinical placement design, implementation, and system challenges; challenges of clinical teaching and learning.

### 3.2. Theme 1: Nursing Education Institutional Factors

Participants reported challenges occurring in nursing education institutions (NEIs) associated with the teaching and learning of clinical competence. These include educational structure, non-prioritization of practical skills such as teaching and learning, unsatisfactory clinical teaching methods and skills, and nurse educator attitude.

#### 3.2.1. Educational Structure 

Some participants felt that the existing educational structure was more focused on theory than practical skills teaching. They believed that the transition of the curriculum from an initial more practice-based preparation to theory-based teaching hampered the learning of practical skills and the development of clinical competence. Moreover, some students were of the view that there was a disconnect between content areas contained in the curriculum and that were taught in the NCs and what actually occurs in the clinical setting:

*“Maybe the problem is coming from our education system, how our educational system is being structured is not helping us, the classroom is more of theory, theory, theory, since we reopened school, we’ve not done one practical at the skills laboratory, which is very bad, is theory, theory, theory, but the work is actually practical, so I prefer the practical should be more.” *

(Third-year student S3STN3).

*“Now when you also come to the school, there you will realize that most NCs they have moved from practical aspect, and they are now focusing on the theoretical aspect.”*

(Post-registration nurse NPRN6).

#### 3.2.2. Non-Prioritization of Practical Skills Teaching and Learning

Compounding the issue of curriculum inadequacy is the failure of nursing colleges to prioritize practical skills teaching. The non-prioritization of practical skills teaching and learning was often manifested in poorly-planned skills laboratory sessions, under-resourced skills laboratories, skills laboratory restrictions (e.g., limited opportunity to practice, limited time, difficulty in accessing skills laboratories, inadequate skills laboratory exposure), overloaded activities, and large student numbers. One of the participants, for example, described the impact of large student numbers:

*“The students are very plenty, we are too much, when you go for practical in the skills lab, to the extent that at times you don’t even see what they are doing when you are forced to stand at the back because of the huge numbers.”*

(Third-year student S3STN3).

In both the school and the CPS, large student enrollments and the resulting effect of overcrowding generated an unfavorable learning environment and limited learning opportunities for students. This problem appeared more pervasive in the school setting since most schools lacked adequate skills laboratory space. Aside from the large number of students, the short time allotted for skills laboratory sessions in the school setting further limited students’ opportunity for hands-on practice, impeding the achievement of learning objectives. Students perceived the time allocated for skills laboratory sessions to be inadequate for the teaching and learning of practical skills.

*“Concerning the challenges in our skills lab, first of all, let’s start with the overcrowding, because of our huge numbers, one class can have sixty plus students entering a small skills lab, no place to sit like my brother said always standing.”*

(Second-year student N2STN7).

*“…the time we have for the skills laboratory within a week is too short, one hour for skills laboratory, that one hour is not really helping us. Because most of us don’t get the opportunity to practice.”*

(Third-year student S3STN2).

Though more prevalent in the clinical learning environment, the non-availability of resources was also a challenge in the school setting, posing a barrier to the teaching and learning of practical skills in the skills laboratory. Participants reported the lack of equipment and essential supplies in the skills laboratory contributing to the limited opportunity to practice. In an attempt to overcome the challenges of inadequate resources in the skills laboratory, some nurse educators forbade students from touching or using certain equipment in the skills laboratory, fearing they would spoil. Other nurse educators simply implemented a policy of “breakages are payable” (post-registration nurse NPRN6) requiring students to pay for items they may have damaged. The policy of forcing students to pay for things they may have damaged in the skills laboratory during the course of learning contributed to student truancy. Some students avoided the skills laboratory for fear of damaging equipment and having to pay for it.

*“Sometimes even if we go to the skills laboratory to learn those procedures, because the equipment is not available it will be very difficult for us to learn it.”*

(Third-year student N3STN6).

*“You can’t even go there because you are even scared to go and spoil something, because here if you spoil anything you will buy.”*

(Second-year student S2STN1).

Despite students’ enthusiasm to engage in deliberate practice to hone their clinical competence, the inaccessibility of the skills laboratory remained a major stumbling block. Some participants noted that the skills laboratory was usually locked up, making it impossible for students to practice on their own. In spite of the restrictions, some participants thought that the skills laboratory provided them with a better opportunity to learn nursing procedures the right way, as opposed to the CPS.

*“Another challenge is that sometimes you will want to go to the skills lab…and then you realize that it is locked, then you can’t find the skills lab tutor to open it for you to learn.”*

(Third-year student N3STN1).

*“…is the skills lab that is helping us to learn the practical properly … when we go there, we normally learn the right things as compared to when we go to the wards.”*

(Third-year nursing student N3STN2).

#### 3.2.3. Unsatisfactory Clinical Teaching Methods and Skills

For the successful teaching of clinical skills, students expected nurse educators to be knowledgeable and competent in clinical skills and student-centered teaching strategies. However, some participants have described the clinical teaching skills of some nurse educators as inadequate, which they believed was detrimental to the development of clinical competence. Additionally, some participants were dissatisfied with teaching strategies, such as lectures and demonstrations, used by some nurse educators in the teaching of clinical skills. The participants thought that such teaching methods promoted memorization over mastery of clinical skills, derailing student confidence.

*“We expect him to know each procedure he’s taking us through.”*

(Third-year student N3STN6).

*“… the tutor will come, project what we are supposed to do and be reading the slides for you, but you know this is something you have to see, this is something that you have to handle but the person is reading it so that you will also follow.”*

(Post-registration nurse NPRN6).

To compliment for the unsatisfactory clinical teaching methods and skills, students and post-registration nurses shared their experiences in the use of deliberate practice, peer learning, and videography to enhance the development of clinical competence. Some students, however, lamented the difficulty in finding contextually relevant videos to aid in student learning when using videography.

*“…what helps us in transferring the knowledge we received in the classroom into practical skills is that we do a lot of peer-tuition.”*

(Third-year student M3STN1).

*“Regarding the videos, the video tutorials we watch, they are mostly in a different environment or usually setting the international world. So, there is always a vast difference. Sometimes the equipment they use to do it, we doubt that we have them in Ghana here but, we always just want to see how it is done so that we will just have an idea or a view of how that procedure is done.”*

(Third-year student N3STN1).

#### 3.2.4. Nurse Educator Attitude

Beyond the unsatisfactory clinical teaching methods and skills, nurse educator attitudes such as anger, domineering, poor communication skills, and lateness were identified as variables that hampered the teaching and learning of practical skills. To bolster the point, a student recounted a personal encounter with nurse educator rage:

*“Some of the tutors come to the class with their own issues from home, so they will come and bring their issues on you, like anger.”*

(Second-year student S2STN5).

### 3.3. Theme 2: Clinical Placement Design, Implementation, and System Challenges

Clinical placement design, implementation, and system challenges such as poor planning of clinical placement, system issues, and the theory-practice gap in the atmosphere of nursing education institutional factors contributed to students’ inability to develop clinical competence smoothly.

#### 3.3.1. Poor Planning of Clinical Placement

The existing clinical placement design and implementation structure in which students were placed in clinical settings for a period of two weeks during the semester according to participants, was inconvenient and bothersome and did little to support the development of clinical competence. Furthermore, participants expressed their dissatisfaction with the absence of pre-assessment of the clinical sites by nursing faculty before clinical placement, claiming that this resulted in students being assigned to clinical sites with limited learning opportunities, thereby hindering the development of clinical competence.

*“…we go to the ward and then do like two weeks practical, when we come back learning becomes difficult for us, so for me, I think when we come to school, we should just focus on the theory and then do the practical when we go on vacation.”*

(Third-year student S3STN5).

*“…the hospitals that they send students to go and learn, the schools should do much research on the hospitals to find out whether the hospitals have the equipment that can aid student learning. The school may send you to a district hospital and over there you go and maybe a day they wouldn’t even have patients in the hospital. You will just be sitting there until it is time for you to close then you close and go.”*

(Third-year student N3STN6).

To make the experience more challenging, students were randomly assigned to wards due to the lack of clear learning objectives for a clinical placement, resulting in inadequate sequencing between students’ year level, content learned in school, and the required objectives to be accomplished in the CPS. However, some participants believed that challenges with large student numbers, rather than a lack of learning objectives, were a primary contributory factor in the random distribution of students to the wards. Moreover, when learners were given learning objectives, noncompliance with these learning objectives in the CPS was another issue that students had to deal with. A post-registration nurse expressed with worry the non-compliance with learning objectives in the CPS:

*“Most often too, what you learnt that you need to put to practice is not where you get to be posted to, a typical example is, you learnt pediatric nursing and you go and you have been sent to the labor ward.”*

(Third-year student M3STN3).

*“It is quite unfortunate sometimes we get there and because all of us cannot be contained in a particular ward, we have to be distributed to other wards.”*

(Third-year student M3STN2).

*“We go with our competencies and at the end of the day sometimes, after your clinical you haven’t even done one procedure from the competencies, its other things you are doing.”*

(Post-registration nurse NPRN3).

#### 3.3.2. System Issues

Some participants felt that if system challenges associated with failed monitoring and poor collaboration had been addressed, the random distribution of students to wards and nonconformity with learning objectives in the CPS may have been prevented. Participants believed that these problems perpetuated because faculty members were unconcerned about student training. This was expressed in the voice of a post-registration nurse:

*“…because our school do not care, the only thing they do is they will give you the competencies you will go on clinical and at the end of the day they will be expecting you to bring back your book signed but, if the school is there they are monitoring, they have sat down with the hospital, my students are coming because of this procedure in Obstetric and Gynecological ward, so please make sure you send them to O and G block so that they can learn what is on the competencies, that one is going to help them.”*

(Post-registration nurse NPRN3).

System inadequacies, such as the failure of regulatory agencies (NMC and MOH) to produce a unified standard procedure manual to standardize practical skills teaching, were seen as contributing to the theory-practice gap, thereby hindering the clinical development of competence. With the lack of an approved standard procedure manual, a confused student struggles to decide which approach to adopt when carrying out nursing procedures. To further compound the problem, the postings of post-registration nurses for mandatory internships were delayed. The delayed postings caused most of them to forget their practical skills.

*“Let me take the cardiac bed for instance, some books will say you should use four pillows, and some will say five. We don’t even know which one to stick to. This person will be reading different thing and I will also be reading a different thing. We will end up confusing ourselves. So, it makes it difficult for us to actually practice what is right.”*

(Third-year student N3STN8).

*“…after completion, our postings delay, we like this, we sat home for almost a year before we were posted for our national service, and you know, nursing is a practice profession, when you sit home for that long without practicing and you are posted later, you turn to forget most of the things, it is hurtful.”*

(Post-registration nurse NPRN 7).

#### 3.3.3. Theory-Practice Gap

Inconsistencies between theory and practice confused students, which further complicated their experiences. Contextual variations in the educational and clinical learning environments, associated with inadequate collaboration between nurse faculty and clinicians, and poorly organized clinical placements, contributed to the theory-practice gap. The ideals taught in classrooms and skills laboratories were not always followed on the wards. In order to integrate into the wards properly, post-registration nurses were forced to abandon the standards taught in schools and approved by the Nursing and Midwifery Council (NMC) in favor of the traditional ways used by nurses in the wards. One student described this as the “learning and unlearning” of ideals. Not only was there a contextual difference between the schools and CPS, but there was also a misunderstanding amongst nurse educators in the teaching of practical skills. Some students noted that nurse educators carried out nursing procedures in varied ways, resulting in confusion among students within and between schools. With a strong voice, one post-registration nurse expressed her dissatisfaction with the situation and considered clinical placement as a waste of time for students:

*“So, looking at what is happening in our hospitals or wards right now, sending students for clinical is waste of their time because how things are done in the ward is different from what is being taught in the classroom.”*

(Post-registration nurse NPRN 7).

*“So, I feel like now we actually learnt and now we are unlearning because some skills that you have or some knowledge that you have you can’t put them in practice because they will tell you that you just can’t practice it because that is what is available for them to practice so you also just have to go through the trend.”*

(Post-registration nurse NPRN7).

*“I have passed through this problem before during practical, what I did was what the man taught me, but the other person said it was not like that, it is different. So, I got angry, very, very angry and the tutor walked me out.” *

(Second-year student N2STN2).

### 3.4. Theme 3: Challenges of Clinical Teaching and Learning

Despite the fact that clinical placement experience was perceived to provide students with a hands-on opportunity to practice, a combination of nursing education institution factors and clinical placement design, implementation, and system challenges contributed to the introduction of clinical teaching and learning challenges such as resource challenges; lack of support; restrictions; exploitations, violence, and fear; personal inadequacy; learner attitude, resulting in the poor development of clinical competence.

#### 3.4.1. Resource Challenge

The apparent lack of resources in both the school and the CPSs contributed greatly to the apparent lack of clinical competence. Both settings lacked critical equipment and materials that may have helped students to learn and provide better patient care. The clear lack of resources in the CPS contributed to the clinicians’ use of conventional ways in patient care, exacerbating the theory-practice gap and having a negative impact on clinical competence development. Students believed that clinicians’ noncompliance with the standards of the performance of nursing procedures in the CPS was due to a lack of resources. In order to overcome the challenge of non-availability of resources, clinicians and nurse educators improvised with what they had on hand, preventing students from experiencing the ideals of the procedures.

*“The more you practice the better you become, unfortunately, most at times we don’t even get, the material to do the right thing, so we always end up doing the wrong thing at the clinical site.”*

(Second-year student M2STN2).

*“I think it’s as a result of insufficient equipment that is why maybe the staff use “shortcuts” in performing nursing procedures in the ward.”*

(Second-year student M2STN3).

*“We have to improvise or we have to imagine that the item is there and we are using it and nursing is not about imagining or improvising.”*

(Second-year student M2STN1).

#### 3.4.2. Lack of Support

Another issue that participants faced in the CPS was a perceived lack of support. According to participants, clinicians were unwilling to teach post-registration nurses and students in the CPS. Both students and post-registration nurses complained about being left on their own and not given enough support during clinical and service placements. In overcoming clinicians’ obvious unwillingness to teach students and post-registration nurses in the CPS, some students made friends with some clinicians in an attempt to lobby them to be taught, while some participants simply resorted to trial and error when performing patient procedures, putting patients in danger. Notwithstanding some participants’ experience that the clinicians were unwilling to teach them, others held a contrary opinion, indicating that a few clinicians were willing to teach them, but the problem was student unwillingness to learn.

*“… the unwillingness of the staff nurses to teach us, you see the thing, they are not willing to teach at all.”*

(Second-year student N2STN7).

*“I have specific wards where I have made friends, so whenever we go to that hospital, I usually make sure that when they are to draw the timetable, I will lobby with them to partner me with those people on shifts. When I am running shift with them, I learn a lot.”*

(Second-year student N2STN5).

*“…when you come to the ward, we are all doing trial and error, so if it works it works, because we have to get it and that is what we are doing.”*

(Post-registration nurse NPRN6).

#### 3.4.3. Restrictions

Aside from the lack of support, participants complained about ward restrictions that prevented them from performing some nursing procedures. Students reported of clinicians preventing them from carrying out certain nursing procedures they deemed as advanced, limiting them to only basic procedures. These restrictions were thought to be more common in bigger facilities, such as teaching hospitals. Coupled with ward restrictions, participants reported being unable to experience the realities of clinical practice due to patient resistance. Some patients refused for students to carry out nursing procedures on them. A student described her experience with patient resistance with a bitter feeling:

*“…if you are a student all the time vitals, that’s all. Medication is done by the staff and the vitals students, documentation and stuff students. But when it comes to the actual procedures like medication administration and wound dressing, they won’t even allow you the student to come near.”*

(Third-year student N3STN8).

*“I remember I was at one ward and I got there and the patient was like why? Is it because there are no staff in the ward that is why they are assigning a student to her? She even sacked me from the room that I shouldn’t come near her, it was very bad.”*

(Third-year student S3STN4).

#### 3.4.4. Exploitations, Violence, and Fear

Participants reported that they were used as extra working hands and expected to perform full-time duties, as if qualified staff, which neglected the accomplishing of their learning objectives. They claimed that clinicians often left the work for them to perform while idling. According to some participants, this was more pervasive in the regional and district hospitals. Moreover, using students to accomplish personal duties, which had no relation to clinical work such as running errands during clinical learning hours, was another challenge that confronted students in the CPS. Students reported they were often sent on errands, making it impossible for them to accomplish the learning objectives.

*“They leave the work in our hands then they will be sitting at the nurses’ station, is very bad.”*

(Second-year student S2STN3).

*“Immediately staff see students in the ward they begin to make you run errands. You can run errands for the whole shift. So, you wouldn’t even have time to learn what you are supposed to learn. Sometimes you go to the ward then a staff gives you a motor bike to go to town and buy food, which is very far, by the time you are back another one is sending you to go and buy fuel, by the time you are back your shift is over.”*

(Third-year student N3STN1).

Stringent clinical learning environment demands, such as forcing students to bring a box of gloves or pay a fee in exchange for access to the CPS, have been highlighted as contributing to student absenteeism in the CPS. Patient resistance and student absenteeism were linked to clinician bullying behaviors such as yelling and the scolding of students and post-registration nurses in the presence of patients and their relatives. Participants reported having been scolded, harassed, and yelled upon by clinicians often times for no apparent reason. Moreover, students were fearful that their attempts to perform procedures in the CPS may endanger the patients. Clinicians’ threats of disciplinary action against students for errors in the delivery of nursing care heightened these anxieties, restricting their ability to achieve learning objectives.

*“…some hospitals demand gloves, you have to buy gloves before you are accepted into the hospital on clinical placement. We are students, we are not people who have salary, so why that policy? In this corona era, one box of gloves cost over GH* *₵50, so you are asking a student each to produce one box of gloves why.* *”*

(Second-year student N2STN3).

*“…they will be shouting on you and they will let every patient know that you are a student, that’s the bad aspect of nursing, so, at times when you go to the patient’s bedside, they will be like are you a student, some will even tell us that they won’t allow us to work on them.”*

(Third-year student S3STN1).

*“Sometimes you are eager to do something for the patient but they will tell you if you do something and it goes wrong, they will hand you over to the appropriate quarters so that they would deal with you. So, in these instances, it put a lot of fears in us the students to come out of our shells and do something.”*

(Third-year student M3STN1).

#### 3.4.5. Personal Inadequacy

When fear was not the major factor, personal inadequacies, such as difficulties in performing nursing procedures in the CPS, perceived inadequate knowledge, and a lack of confidence, were noted as demotivating in the pursuit of clinical competence. One student, for example, described the difficulties in performing nursing procedures:

*“When you are being asked to say it verbally, you will do it or if you are being asked to write it, you will do it. But here is the case, do it let me see so that I can score you, then you find yourself wanton because you don’t know the equipment.”*

(Second-year student M2STN3).

#### 3.4.6. Learner Attitude

Students also reported that the attitude of some of their colleagues such as truancy, the lack of seriousness, and unwillingness to learn, had a negative impact on their clinical competence development in the CPS. Despite the readiness of some clinicians or nurse educators to assist students to learn, some students were just not willing to learn.

*“Some of the student too because of their attitude they tend to run away and not practice or learn anything from the ward.”*

(Third-year student S3STN5).

## 4. Discussion

Despite the fact that nursing education in Ghana is undergoing transformation, findings from this study suggest that the curriculum used by nursing colleges is not in synchrony with happenings in the CPS, implying that it is poorly tailored to meet the actual needs of patients in the practice setting. Moreover, our findings suggest that the sudden transition of the curriculum from practice-based to a theory-based teaching impeded the learning of clinical skills and the development of clinical competence. Some participants were also not happy with the block model of clinical placement used in the study setting. Most were simply not happy with the distortion of classroom work with this model. Findings of the study also revealed a number of factors that hindered the development of clinical competence in the CPS. For example, most participants were dissatisfied with the general lack of resources and support for learning in the CPS.

Our findings are similar to what has been reported in previous studies. Salifu et al. [13] in their study to explore the experiences and perceptions of the theory-practice gap in nursing education in a resource-constraint setting, found that more theory-based teaching impeded the development of clinical competence. Arguably, the block and integrated models of clinical placement are the most widely used in nursing curricula [36]. With the block model, students are placed in CPS for a period of two to four weeks during the semester [37]. The integrated model involves a semester-long blend of academic and clinical learning, with a day or two set aside each week for clinical placement experience [36]. Comparative analysis of the benefits of the two models has been varied, inconsistent, and inconclusive. What is clear is that the concerns about the use of the block model in this study setting had nothing to do with its effectiveness in promoting clinical competence development, but rather with comfort issues. In a critical review of block and integrated practice learning models, Coleman [36] confirmed that while the block model provided enough time for students to concentrate on clinical learning without interference from classroom work, it was perceived to contribute to the theory-practice gap. Indeed, with the use of this model, students were more at risk of being used as extra hands rather than learners with learning objectives to accomplish. Consistent with Coleman’s view, the use of the block model in this setting could have accounted for the use of students as extra hands.

However, this could be avoided with much involvement and collaboration between NEIs and CPS. Students’ learning objectives and clinical competencies to be attained during clinical practicum should be explicitly stated and shared with the practice sites prior to clinical placements. Moreover, similar to Salifu et al.’s [13] finding, no form of assessment was usually undertaken by school authorities before students were sent for clinical placement, resulting in students being placed in practice sites without the requisite capacity and opportunities available to support student learning. In addition, similar to Killam and Heerschap’s [38] finding, short clinical placement duration impeded the development of clinical competence. Professional opinions and studies tend to favor longer clinical placement duration for nursing and other health related programs [36], but none is definite on the exact duration or clinical hours that is ideal for clinical placement [36]. A re-examination of the needed classroom contact hours before clinical placement and assessment of the clinical sites prior to clinical placements is required.

From the experiences of participants in this study, there was no formal support structure to facilitate student learning in the CPS. Students and post-registration nurses were left to practice as qualified staff without supervision, thereby endangering the lives of patients and hindering the attainment of clinical learning objectives. This finding reflected the findings of Begley [15] and Salifu et al. [13], in which the authors confirmed that students were often left alone without support to navigate the complex and challenging circumstances of the CPS. Where the assumption of full professional responsibilities by students and post-registration nurses was not a problem, sending students on errands by clinicians was another factor that confronted students in the CPS in this study. Salifu et al. [13], Jamshidi et al. [14], and Killam and Heerschap [38], in their studies, also reported that students were often sent on errands to the detriment of their learning in the CPS. The cumulative effect of all of these challenges confronting the development of clinical competence is an incompetent nurse in practice. Such nurses serve as a threat to patient safety and care outcomes with the provision of suboptimal care [9,39]. Due to the lack of knowledge and inability to carry out nursing procedures, students and post-registration nurses in this study felt they were ill-prepared for the demands of CPS, similar to Begley’s finding [15]. Further, students were afraid that their attempts to perform procedures in the CPS may endanger the lives of patients. This often precludes students from carrying out procedures they deemed as advanced in direct patient care. This fear was usually compounded by the negative attitude of nurse educators and clinicians. The literature is rife with the negative attitude of both nurse educators and clinicians, which appears to serve as a threat to student learning [8,14,40]. In this study, the negative attitude of clinicians towards nursing students made them uninterested in learning in the CPS.

Given that today’s students and post-registration nurses are tomorrow’s nurses, there is the need for stringent policy directives to resolve the current problems faced by students and post-registration nurses in their attempt to develop clinical competence. Curriculum reform is required to improve the quality and effectiveness of nursing education in the study setting. Such curriculum reforms must take into account the local contextual intricacies of the CPS, as well as customized student outcomes [41]. This will assist students and post-registration nurses in gaining the basic requisite competence to be relevant in the health facilities where they will be practicing after graduation. Some countries have also introduced compulsory transition programs with formal support and education for newly qualified nurses (NQN) [42]. However, despite the introduction of a compulsory transition program in the form of internship for NQN in Ghana, there has not been any formal support systems to accompany it. Although the Nursing Council expects that NQN within the internship period are supervised by experienced nurses, the NQN assume full professional roles and function as such without adequate supervision. In other parts of the world, the responsibility of facilitating teaching in the clinical learning environment is a shared one between NEIs and the CPS [12,15]. The introduction of a formal support system, such as a preceptorship or clinical placement coordinators appointed from the clinical site to oversee the activities of preceptors and students, promises to be an effective strategy to promote student learning and the development of clinical competence [13,15]. This could help bridge the theory-practice gap and ease the challenges associated with the development of clinical competence. Moreover, given the numerous challenges that face nursing education and the development of clinical competence in this study setting, a combination of clinical placement experience; immersive, student-centered, and experiential teaching and learning strategies; and simulation-based clinical nursing education could be more effective approaches to nursing education. The use of simulation pedagogy to complement clinical placement experience is regarded to be more effective in supporting student learning and clinical competence development [1,22,23]. However, for simulation pedagogy to flourish in a low-resource setting, its design, implementation, and evaluation need to be guided by a framework that is tailored to match the local needs and resources of the setting.

### Study Limitations

The study’s participants were recruited using a purposive sampling technique, which could have resulted in sampling bias. The researchers, however, utilized an independent person in the recruitment process to reduce the sampling bias. Similarly, restricting eligible participants to those with access to computers, tablets, or phones may have resulted in sampling bias. However, persons who did not have such devices but were in close proximity to others who did were encouraged to pair and utilize them in the zoom sessions. Due to time and budget constraints, the study was only conducted in three NCs and their primary clinical sites across the three zones of the study setting. As a result, the transferability of the study’s findings may be constrained by the participants’ and settings’ selections. However, because the NCs all have the same curriculum, academic guidelines, facilities, and clinical sites, the results of this study may appear similar if duplicated in different sites within the same study setting.

## 5. Conclusions

Nursing education will continue to be critical in developing a strong health work-force to achieve global health and universal health coverage. Nursing education must continually improve to stay relevant in light of the complexities, dynamism, advances in technology, and increasing demands of patient needs. Despite perceiving clinical placement experience as beneficial, the totality of findings of this study have shown that the current approach to nursing education in the study setting falls short of been effective. As a result, major reforms in nursing education need to be undertaken to ensure the training of competent nurses with the ability to transition from students to independent clinicians and have a positive impact on patient care outcomes. Curriculum reforms, restructuring of clinical placement, retooling of skills laboratories, infrastructural expansion, and the use of simulation pedagogy in the teaching of clinical skills are all examples of improvements required in nursing education in the study setting. These reforms should be championed through effective collaboration of key stakeholders of nursing education such as the regulatory body, management of NEIs, and the CPSs.

## Figures and Tables

**Table 1:** Topic guide for focus group discussions on clinical competence development.

Tell me more about your experience in the teaching and learning of practical skills in your course of training. Describe the teaching methods used in assisting you to become competent in nursing practice. Describe the role the skills laboratory played in helping you achieve your clinical learning objectives. What challenges (if any) do/did you encounter in the skills laboratory? How do you think the challenges (if any) you mentioned in question four could be resolved? Describe the actions of the tutor that promotes or impedes learning. Describe your experience in integrating theory to practice in the clinical environment. Describe the influence of the clinical learning environment in assisting you to develop skills in readiness for practice. What challenges (if any) do you encounter in the clinical learning environment? Describe ways you think you could be assisted to acquire clinical skills in readiness for practice.

**Table 2:** Demographic characteristics of participants.

Variables	Students	Post-Registration Nurses
**Gender**		
Male	25	7
Female	15	8
**Age (in years)**		
21–25	33	7
26–30	7	8
31–35	-	-
36–40	-	-
41–45	-	-
**Program level**		
Second year	20	-
Third year	20	-
**Zone**		
Northern zone	15	7
Middle zone	12	5
Southern zone	13	4
**Work experience (in months)**		
1–5	-	6
6–10	-	2
11–15	-	7

## Data Availability

The data presented in this study are available on request from the corresponding author.
